# Inheritance patterns of lower urinary tract symptoms in adults: a systematic review

**DOI:** 10.1111/bju.16517

**Published:** 2024-08-26

**Authors:** Lorcan Moore, Sachin Malde, Prokar Dasgupta, Arun Sahai, Nicholas Raison

**Affiliations:** ^1^ Guy's King's and St Thomas' School of Medical Education King's College London London UK; ^2^ Department of Urology Guy's and St Thomas' Hospital London UK; ^3^ Department of Urology King's College Hospital London UK

**Keywords:** lower urinary tract symptoms, heritability, twin studies, genome‐wide association study, BPH

## Abstract

**Objective:**

To compile and evaluate the heritability and inheritance patterns of lower urinary tract symptoms (LUTS) in adult cohorts.

**Methods:**

Searches of five databases (PubMed, Embase, APA PsycInfo, Global Health, and OVID Medline) commenced on 6 July 2024, resulting in 736 articles retrieved after deduplication. Studies evaluating heritability patterns, gene frequencies, and familial aggregation of symptoms were included for review. Screening and predefined eligibility criteria produced 34 studies for final review. A descriptive analysis of synthesised data was performed, adhering to the Preferred Reporting Items for Systematic Reviews and Meta‐Analyses guidelines. The Cochrane Risk of Bias in Non‐Randomised Studies of Interventions (ROBINS‐I) tool and the Johanna Briggs Institute checklist were used to evaluate these studies.

**Results:**

Ten of the 34 studies (29%) described general LUTS, 14 (41%) described symptoms due to benign prostatic enlargement (BPE), nine (26%) described urinary incontinence (UI; urge UI [UUI], stress UI [SUI] and mixed UI [MUI]), four (12%) described nocturia alone, two (6%) described overactive bladder (OAB), and four (13%) described other specific symptoms (frequency, postvoid residual urine volume). BPE symptoms, UI (MUI and UUI), nocturia alone, and frequency alone were associated with genetic predisposition, whilst OAB and SUI had more modest inheritance.

**Conclusion:**

The pathogenetic and pharmacological mechanisms fundamental to LUTS manifestation are highly heterogeneous. Further work is required to evaluate the inheritance patterns of LUTS more extensively.

**Abbreviations**
BPE: benign prostatic enlargement
DZ: dizygotic
GWAS: genome‐wide association studies
JBI: Johanna Briggs Institute
MUI: urinary incontinence
MZ: monozygotic
OAB: overactive bladder
OBND: oblique bladder neck descent
OR: odds ratio
PRISMA: Preferred Reporting Items for Systematic Reviews and Meta‐Analyses
PRS: polygenic risk score
PVR: postvoid residual urine volume
ROBINS‐I: Risk of Bias in Non‐Randomised Studies of Interventions
SNP: single‐nucleotide polymorphism
SUI: stress urinary incontinence
SWiM: Synthesis Without Meta‐Analysis
UI: urinary incontinence
UUI: urge urinary incontinence

## Introduction

Lower urinary tract symptoms (LUTS) are a common and significant source of morbidity, however, our understanding of their pathophysiology remains poor. Current understanding suggests a complex multi‐faceted aetiology, although this is yet to be effectively consolidated. A role for genetic factors is supported by evidence of familial aggregation. Stress urinary incontinence (SUI) has been shown to cluster amongst family members of affected individuals [1], whilst early‐age onset of benign prostatic enlargement (BPE) has a genetic susceptibility that demonstrates a Mendelian dominant inheritance pattern [2].

Numerous other variables are also involved in LUTS pathogenesis, including modifiable lifestyle risk factors and environmental causes [3]. Causative mechanisms are likely to be convoluted and multifactorial.

Inheritance patterns in LUTS have not been extensively studied, leaving our understanding limited. However, gaining a deeper insight into heritability and the influence of environmental factors is crucial for comprehending LUTS and accurately characterising individual variations. Identifying genetic factors associated with LUTS can reveal genes involved in their development, potentially leading to the identification of targets for gene therapy. This knowledge can guide more personalised treatment approaches, tailored to each patient. Assessing heritability can be achieved through a variety of study designs.

Twin studies play a crucial role in clarifying the interplay of genetic and environmental factors on phenotypes, which is achieved through comparisons of variation between monozygotic (MZ) and dizygotic (DZ) twins. Heritability studies, structured to examine genetic influences at a population level, amalgamate all genetic factors for efficient measurement. The classic twin design approach dissects phenotype variations into additive genetic variance, common factors, and environmental influences. However, as a standalone method for estimating heritability, twin studies do not provide insights into specific genes, their individual strengths, or the number contributing to a phenotype—known as the genetic architecture.

Recent advancements in sequencing technologies have revolutionised the accurate genotyping of individuals with LUTS. Notably, candidate gene studies and genome‐wide association studies (GWAS) have emerged as prominent methodologies for elucidating the genetic architecture of specific traits or pathologies. GWAS, for instance, have successfully identified novel loci and common variant sites associated with type 2 diabetes, offering benefits in surveying diverse populations and guiding candidate drug investigations [4].

However, GWAS are limited by the need for large samples [5]. Despite this, both GWAS and twin‐study approaches can complement each other by addressing issues such as population stratification and gene–environment correlations [6]. Thus, these methodologies collectively offer valuable insights applicable to prognostic stratification, understanding therapeutic responses, and identifying new pharmacological targets for treating LUTS.

The existing literature base of LUTS heritability, as a combined entity, is limited, therefore the aim of this review was to explore the candidate gene polymorphisms, inheritance patterns, and familial aggregation in association with LUTS in adult populations.

## Materials and Methods

### Eligibility Criteria

The inclusion criteria for studies in this review comprised:

- Adult population (age >18 years) of either sex;
- Assessments of heritability or genetic factors (e.g., polymorphisms such as SNPs or deletions) potentially associated with LUTS;
- Explicit LUTS diagnosed via all appropriate methods, including diagnostic questionnaires, uroflowmetry studies, clinical evaluation, histology, patients on medications (in the case of alpha blockers for BPE) or documented interventional therapy;
- Original publications in any language, irrespective of publication date.

The specified exclusion criteria were:

- Histological diagnoses in the absence of symptoms;
- Asymptomatic screening patients;
- Recurrent UTIs;
- Anatomical urinary abnormalities;
- Concurrent stone disease;
- Heritability measures with sole reference to urinary microbiota;
- Case series and case reports.

### Search Strategy

The databases PubMed, Embase, APA PsycInfo, Global Health, and OVID Medline were utilised to retrieve relevant articles on 6 July 2024. Search strategies were conducted according to the Preferred Reporting Items for Systematic Reviews and Meta‐Analyses guidelines (PRISMA [7]). Published studies eligible for inclusion were chosen from the commencement of each database up until 6 July 2024. Data S1 contains a full list of the search terms used.

### Screening and Data Extraction

After removal of duplicates, the initial search retrieved 736 studies. Titles and abstracts were screened, and studies not fulfilling the eligibility criteria were removed. Thus, we commenced full‐text analysis of 73 studies.

**Fig. 1:**
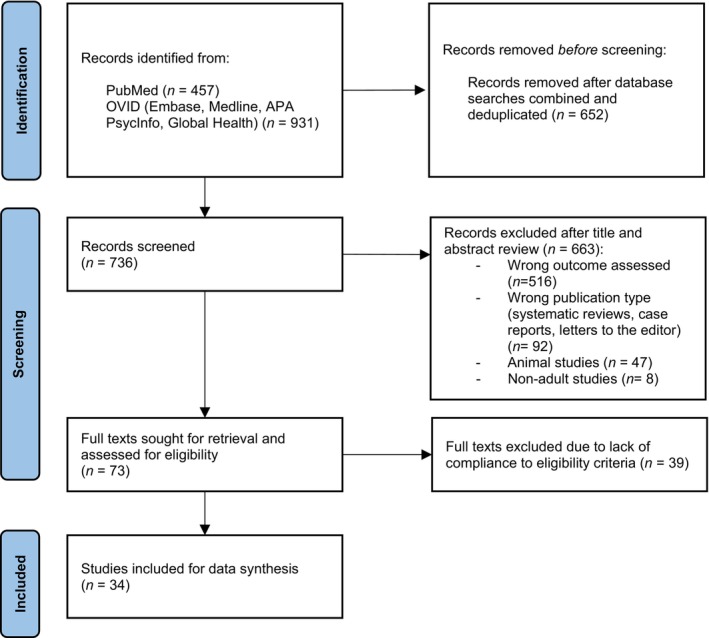
Preferred Reporting Items for Systematic Reviews and Meta‐Analyses diagram defining the process of search strategy.

Based on the predefined data collection criteria, the following were extracted from each study: first author year of publication, and country; study design; LUTS diagnostic tools; participant information (mean age, standard deviation, gender discrepancies); odds ratios (ORs); and outcome measures and key study findings. Figure 1 shows the search strategy and screening procedure.

### Quality Assessment: Risk of Bias

To assess risk of bias within publications, we employed the Risk of Bias in Non‐Randomised Studies of Interventions (ROBINS‐I) methodology [8], consisting of pre‐intervention, at‐intervention, and post‐intervention domains. The premise of this tool is a comparison between an observational or case–control study and a hypothetical model randomised controlled trial. Studies are graded at segmental bias thresholds: low, moderate, serious, and critical; judgements from each domain confer an overall risk score for the intended outcome being assessed [8].

Because twin studies have a lack of theoretical randomisation, a further quality assessment was employed: the Johanna Briggs Institute (JBI) critical appraisal checklist for case–control studies [9]. This quality assessment measure synthesises risk assessment domains including confounding variables, measurement of exposures/ cases, case matching, and statistical analytical methods. Review and reporting of the risk of bias is compatible with the PRISMA protocol recommendations [10].

## Results

### Evidence Synthesis

After screening, 34 studies were included for analysis. Studies were grouped according to LUTS: 10 studies specified heritability of LUTS subtypes and combined LUTS subcategories; 14 reported BPH symptomatology and BPE findings; six reported inheritance of urge urinary incontinence (UUI); seven described SUI; two reported mixed urinary incontinence (MUI); two focused on overactive bladder (OAB); and two additional studies specified other symptoms (oblique bladder neck descent [OBND]; postvoid residual urine volume [PVR]). Due to high heterogeneity of the included studies, descriptive data synthesis was performed in accordance with the Synthesis Without Meta‐Analysis (SWiM [11]) reporting framework. Table A1 summarises the list of SNPs and associated genes implicated in LUTS subcategories.

### Composite LUTS

Ten studies evaluated the heritability of LUTS in general, including men and women. The majority were cohort studies with average patient ages of over 50 years (Table S1). All included studies used validated questionnaires including the IPSS, AUA and International Continence Society (ICS) symptom indices to stratify levels of symptoms. Nine studies confirmed presence of heritability, whilst one found no association [14]. Three studies reported heritability of symptom scores, with additive genetic factors accounting for 37%–63% of variation [34, 35, 36]. All three studies used the IPSS to assess LUTS. Rohrmann et al. [35] additionally determined that 72% of LUTS/ BPH variability in moderate to severe cases (defined by the authors as IPSS >8) was accounted for by genetic factors, whilst the study by Yue et al. [37] suggested heritability of 40.48% for mild to severe symptoms. Evidence from four twin studies reporting composite LUTS (primary outcome was assessment of storage symptoms) showed higher concordance rates and tetrachoric correlations (a measure of association between non‐binary variables: LUTS subtypes and zygosity) of general LUTS in MZ twins [35, 38]. Urinary incontinence (UI) frequency and nocturia displayed the strongest genetic association with LUTS. These studies also demonstrated a linear increase in LUTS with age [34], as well as higher rates of incontinence in female cohorts. Findings from Afari et al. [36] revealed that, utilising the best‐fitting models, none of the influence arose from shared environmental factors.

Four GWAS showed that SNPs on chromosome 8 were protective of LUTS symptoms when adjusting for age and the presence of additional genetic variants (OR 0.78) in African‐American men. Additional studies demonstrated associations between SNPs and LUTS severity [26, 32] (OR 2.21 for *TREK1* SNP [26], 0.553 for *IL28‐Rα* SNP [32]). The studies analysed spontaneous polymorphisms in genes associated with the inflammatory response underlying BPH progression, and genes encoding aberrant potassium channels within overactive detrusor muscle. One additional SNP was deemed protective of LUTS and was associated with lower IPSS [21]. These GWAS therefore implicate a range of genes in LUTS pathogenesis, both protective and risk factors.

### Symptoms Secondary to Benign Prostatic Enlargement

Fourteen studies explicitly assessed genetic associations with BPE symptoms; 10 studies showed significant association between SNPs and symptoms of prostate enlargement, and familial aggregation (Table S2). Analyses of SNPs, including *LILRA3, TLR4* and *PGR*, outlined increases in OR (1.34 [24], 2.96 [28] and 1.36 [25], respectively) of BPE symptoms, observed in Chinese cohorts. A UK Biobank‐based GWAS confirmed an increased OR for SNPs at *PGR* (OR 1.36), whilst additionally revealing two SNPs protective against prostate enlargement symptomatology [25], nearby other genes including *MPPED1* and *NPAP1* (OR 0.72 and 0.66, respectively). Li et al. [25], Gudmundsson et al. [19] employed appropriate significance thresholds and approximate conditional analyses to investigate any additional signals at the loci determined to be significant at a genome‐wide level for benign hyperplasia; this identified other association signals. Hellwege et al. [22] traced susceptible loci for BPH symptom manifestation to chromosomes 5 and 6, containing loci at *SYN3* and *SORCS1*, which are neuronal and transmembrane proteins, respectively. In contrast, SNPs associated with DNA repair and inflammation showed insignificant association with BPH symptoms [23, 31]. Similarly, genes involving testosterone metabolism were found not to be correlated with IPSS or maximum urinary flow rate values [15]. Nevertheless, Gudmundsson et al. [19] revealed correlations between PSA and polygenic risk score (PRS); every standard deviation increase in PRS was associated with an 8.6% increase in PSA levels (with joint conditioning of prostate cancer).

With regards to twin and case–control studies, two reports suggested codominant or autosomal dominant patterns of inheritance [12, 39]. Statistical analysis by Pearson demonstrated a cumulative risk effect: in those with first‐degree relatives with symptomatic BPH, one first‐degree relative conferred a 1.74 OR, whilst two relatives conferred a 4.74 OR [39], demonstrating familial aggregation effects. However, Gasperi et al. [40] found more moderate genetic contributions to BPH symptoms (20% heritability), with greater emphasis on non‐shared environmental influences (70% heritability) using a multivariate reduced common pathway.

### Storage LUTS, Overactive Bladder and Stress Urinary Incontinence

#### Overactive Bladder Dry and Overactive Bladder Wet

Two studies reported OAB findings, and OAB subcategory findings (wet and dry; Table S3). When OAB wet and dry were combined, there was consensus across both studies that OAB was not a highly heritable trait. The non‐gender‐specific GWAS conducted by Funada et al. [41] determined that OAB heritability (*H* ^2^) was 0.027, signifying very minor genetic effects on OAB heritability. There was also no association between any SNP and OAB, either wet or dry. Instead, this study demonstrated a larger role of environmental factors, particularly shared influences such as learned behaviours from family members, in incidence of familial OAB aggregation [41].

Similarly, a Swedish male‐only twin study, reported minor heritability estimates, in which OAB wet and dry combined had 0.10 heritability [38], meaning minor genetic influences on OAB aetiologies. This cohort was also considerably younger in age than other OAB‐spectrum studies (age range 20–46 years). Subcategories of OAB were also assessed: OAB dry had the highest environmental influence, with only a 0.04 heritability value when confounding for age, body mass index, and smoking history [38].

#### Nocturia and Frequency (Without Urgency)

Reports concerning nocturia demonstrated conflicting results; three studies were twin studies whilst one was a case–control study (Table S3). Three studies showed heritability of nocturia of 21% [36], 22% [34] and 19% [37], whilst the fourth study reported 48% heritability when adjusting for incontinence due to pelvic floor weakness [38]. Conversely, estimates for urinary frequency without urgency in twin studies showed a greater genetic predisposition. Heritability estimates ranged from 36% [36] in the twin study to 71.37% [37] in the case–control study, confounded by age, parity, and BMI.

#### Urinary Incontinence

The general category of UI was detailed in 11 studies, with varying accounts of heritability depending on the UI subtype (Table S3).

##### Urge Urinary Incontinence

Six studies evaluated UUI, of which only one report found no genome‐wide association with UUI [27]; that study investigated specific SNPs through GWAS. Meanwhile, genome‐wide significance was achieved for SNPs such as *HTR2A* [13] (3.06 OR), *EDN1* [16] (1.70 OR), *AGK* [17] (3.21 OR), and *CIT* [30]. In a further GWAS, Cartwright et al. [17] determined that there was a shared genetic determinant of vulnerability to SUI and UUI at *AGK*, an intron variant.

A twin study of 1500 twin pairs found heritability of 34% for UUI [36], denoting only moderate genetic influences on UUI; further analyses suggested that environmental factors alternatively play a large role in UUI manifestation.

##### Stress urinary incontinence

Seven studies investigated SUI, and results were mixed. Four studies suggested high heritability estimates [16, 17, 29, 42]. Reischer et al. [29] found significance at three loci conferring SUI phenotypes, including *SERPINA5* and *MMP1*. Altman et al. [42] concluded that 41% of variations in SUI liability were accounted for by genetics, whilst SUI ORs were between 1.73 and 4.27 [16, 17], signifying large genetic influences on SUI.

Conversely, three studies reported no inheritance influences on SUI. A twin study concluded that environmental factors alone contributed to 77.6% of variance, with genetics supplying only 1.46% [43]; another twin study found no statistically significant difference between MZ and DZ cohorts [44].

Penney et al. [27] utilised GWAS to examine three SNPs with SUI and found no statistical significance, although when accounting for predetermined risk factors, genome‐wide significance of *WDR54* was attained.

A further study investigated OBND, a subset of SUI. OBND was deemed more heritable than bladder neck descent (BND), and ACE modelling demonstrated 59% and 51% heritability influences for OBND and BND, respectively, [45] using a twin study approach.

##### Mixed Urinary Incontinence

Two studies explicitly mentioned MUI; Rohr et al. [44] concluded that 27% and 55% of variation in MUI phenotype were accounted for by genetics in the age groups 46–48 years and 70–94 years, respectively, through a twin‐study approach. However, utilising GWAS, Penney et al. [27] showed no genome‐wide significance for MUI in unadjusted analysis, but nevertheless reported that top results for MUI and UUI subtypes were concordant with one another, suggesting moderate collective inheritance mechanisms for both, attributable to genetic influences.

### Additional LUTS

Other non‐specific symptoms investigated included PVR and weak urinary stream (Table 1). Haga et al. [14] found increased PVR and voiding time in variants of *TRP64ARG* and *ARG64ARG* of the β_3_ adrenoreceptor gene, although this SNP was not significant when analysing LUTS in general.

**Table 1:** Data extraction table summarising studies assessing additional LUTS subcategories, with key findings and risk of bias assessments.

Study information		Population					Results	
Reference/Country	Gene symbol/polymorphism (if applicable) of interest	Specific LUTS diagnosis	LUTS diagnostic tool(s)	Number	Male, %	Mean age, years	Key study findings	ROBINS‐I tool
GWAS								
Haga et al. (2021)/Japan [14]	Variants of β_3_ adreno‐receptor gene: *TRP64ARG* *ARG64ARG*	PVR, composite LUTS	Ultrasonography, PVR, uroflowmetry, ICS pad test	Cases: 129 Controls: 247	Cases: 100 Controls: 100	Wild type: 67.00 ± 5.30 Cases: 67.00 ± 4.90	PVR increased in variant type, and voiding time	
Twin studies								
Dietz et al. (2005)/Australia [45]	–	PVR, voiding	Translabial ultrasonography	46 MZ pairs 24 DZ pairs 38 non‐twins	MZ 0 DZ 0 Non‐twins 0	N/A age range 18–24 years	Approximately 50% of bladder and urethral mobility in nulligravid women is influenced by genetic factors	
Meikle et al. (1999)/USA [34]	–	Weak stream	AUA score, TRUS, DRE	MZ: 83 pairs DZ: 83 pairs	MZ: 100 DZ: 100	MZ: 55.00 ± 12.50 DZ: 54.30 ± 11.90	No evidence of any heritability for weak stream and intermittency	
Case control, candidate gene association								
Yue et al. (2019)/China [37]	–	DRE, ultrasonography	Cases: 94 Controls: 106	Cases: 94 Controls: 106	Cases: 100 Controls: 100	Cases: 64.75 ± 11.75 Controls: 62.24 ± 12.36	Weak stream was less heritable (heritability 2.70%)	

DZ, dizygotic; GWAS, genome‐wide association study; ICS, International Continence Society; MZ, monozygotic; N/A, *mean age not provided*; PVR, postvoid residual urine volume; ROBINS‐I, Risk of Bias in Non‐Randomised Studies of Interventions.

Through twin‐study analysis, Dietz et al. [45] showed that 50% of bladder and urethral mobility had genetic influences; interestingly, this study was conducted in nulligravid women, thereby controlling for a significant confounding variable for pelvic support (parity). Two studies reported values of weak stream; both reports determined that it had a minimal heritable component, with heritability between 0% and 2.70% [34, 37]. These reports utilised twin‐study and case–control methodologies.

### Quality Assessment Results

Table 2 shows the quality assessment results for studies that used both the ROBINS‐I and JBI checklist appraisal tools as outlined in *methods* section.Segmental colour shading outlines thresholds of bias risk, ranging from low to critical. Overall risk‐of‐bias for each study is a compilation of each bias demain, and full tables for each quality assessment methodology can be found in Tables S4 and S5.

**Table 2:** Quality assessment results via ROBINS‐I and JBI Checklist.

Study (year)/ country	ROBINS‐I	JBI Checklist
Abdullah et al. (2018)/Iraq [12]	Serious	
Afari et al. (2016)/USA [36]	Moderate	
Altman et al. (2008)/Sweden [42]	Moderate	
Aniulis et al. (2021)/Lithuania [13]	Low	
Berges et al. (2009)/Germany [15]	Moderate	
Cartwright et al. (2021)/UK, Finland [16]	Low	
Cartwright et al. (2014)/UK [17]	Serious	
Cheng et al. (2020)/China [18]	Serious	
Dietz et al. (2005)/Australia [45]	Moderate	
Funada et al. (2017)/Japan [41]	Moderate	
Gasperi et al. (2019)/USA [40]	Moderate	
Gudmundsson et al. (2018)/Iceland, UK [19]	Low	
Haga et al. (2021)/Japan [14]	Moderate	
Helfand et al. (2013)/USA [46]	Serious	
Hellwege et al. (2019)/USA [22]	Moderate	
Hsing et al. (2007)/China [23]	Moderate	
Jiao et al. (2013)/China [24]	Moderate	
Li et al. (2021)/UK [25]	Moderate	
Meikle et al. (1999)/USA [34]	Moderate	
Na et al. (2017)/USA, China, Finland [20]	Moderate	
Nedumaran et al. (2018)/USA [26]	Serious	
Nguyen et al. (2008)/USA [43]	Moderate	
Partin et al. (1994) [47]/USA	Serious	
Pearson et al. (2003)/USA [39]	Moderate	
Penney et al. (2020)/USA [27]	Moderate	
Qiu et al. (2019)/China [28]	Moderate	
Reischer et al. (2020)/Austria [29]	Low	
Richter et al. (2015)/USA [30]	Moderate	
Rohr et al. (2004)/Denmark [44]	Serious	
Rohrmann et al. (2006)/USA [35]	Low	
Tanaka et al. (2010)/Japan [31]	Serious	
Wennberg et al. (2011)/Sweden [38]	Moderate	
Xiao et al. (2015)/China [32]	Serious	
Yue et al. (2019)/China [37]	Moderate	

JBI, Johanna Briggs Institute; ROBINS‐I, Risk of Bias in Non‐Randomised Studies of Interventions.

A common field identified as higher risk included the pre‐intervention domains, especially baseline confounding. Certain studies, especially those in subjects with BPH diagnoses, did not account for noteworthy confounding variables such as exclusion of prostate cancers [12], A key limitation of certain reports was lack of appropriate case and control matching [44], and missing data on key population metrics or analysis measurements [17, 21]. In total, five studies demonstrated low risk of bias according to ROBINS‐I appraisal, 18 studies had moderate risk of bias and nine had serious risk.

An inherent cause of bias in many of the studies was confounding and lack of randomisation applicable to the study design, especially twin studies; therefore, JBI checklist tools were employed to further evaluate risk of bias. Two studies were designated as having serious risk, 10 moderate, and 20 low. The most common metric identified as serious risk was explanation of confounding variables; whilst many reports listed these variables, there was no explicit mention of remedies, hence there was reporting bias.

## Discussion

The aim of this systematic review was to appraise the existing literature examining the heritability of LUTS in adult populations, using all available evidence with methods including GWAS, twin studies, and case–control candidate gene studies. This is the first report to systematically review LUTS inheritance in adults. The acquired evidence supports a significant genetic influence on LUTS identified with both GWAS and twin studies. GWAS tended to focus on subcategories of LUTS, especially prostate symptoms resulting from BPH, and UI, whilst twin studies evidenced heritability prominently in composite LUTS, with UI as another prominent domain.

We found that LUTS subcategories had heterogeneous levels of inheritance. Studies reporting BPH findings showed significant genetic effects, with the two studies disputing this conclusion being focused solely on two SNPs. Over 32 loci were associated with BPH risk and BPH symptom scores, demonstrating the multidimensional and complex genetic origins implicated in this disease. Prominent loci in GWAS analyses included *LILRA3*, an anti‐inflammatory modulator. This gene has been found in other conditions affecting inflammatory responses, such as inflammatory bowel disease and prostate cancer [48]. As an entity, inflammatory processes are common throughout the LUTS groups; both inflammatory mediators and homeostatic pathways have been suggested as mechanisms for UI (UUI and SUI) manifestion. *SERPINA5* was shown to be involved in inflammatory mechanisms through its regulation of proteolytic intra‐ and extravascular actions [49]. Although these assumptions cannot be deduced from the GWAS findings of this review, they offer another perspective for the pathogenesis of LUTS components such as UI.

In many of the included studies, SUI was shown to be the least genetically determined subcategory of UI. This corresponds to our understanding of the pathophysiology of SUI and its correlation with known risk factors for mechanisms of incontinence such as childbirth (vaginal delivery vs caesarean section [50]). However, there is evidence for the contrary: a systematic review by Cartwright demonstrated a statistically significant pooled effect size (OR 2.1) associated with a polymorphism at *COL1A*, a gene linked to reduced type I collagen, in four studies [51]. This was the only statistically significant SNP associated with SUI when meta‐analyses were performed. One study included in our review disputed the verdicts on *COL1A*, finding no association of *COL1A* with SUI [29], although its sample size was very limited (analysis was only conducted in nine samples). Other LUTS can be better explained by genetics; nocturia, frequency and UUI/ MUI were established more conclusively in MZ versus DZ twins. *DAB1* and *CIT 1*, polymorphisms identified in this review, are implicated in neuronal migration [52], and central nervous system development and bladder contractility, [53] respectively. This could perhaps explain aberrances in the mechanisms of continence and bladder control causing UI. With regard to study design, UUI had high ORs for specific SNPs, vs general heritability estimates gathered from twin‐study investigations.

Many of the studies chosen did not have overlap in the SNPs assessed, therefore, it was not possible to undertake pooled analysis of these polymorphisms. Self‐reported research limitations were reported, including population stratification bias, genotyping methodological errors, and selective outcome reporting. A systematic review of pelvic organ prolapse similarly reported heterogeneity among its included studies, and attributed this partly to discrepancy in case definitions [51]. Two studies, however, reported findings associated with *ADAMTS16* polymorphisms in OAB and UI, with different conclusions as to their association with clinical phenotypes. Richter et al. [30] concluded that *ADAMTS16* conferred higher risk of the UUI phenotype, whilst Funada et al. [41] demonstrated no association with OAB. The role of *ADAMTS16* is still uncertain, although it has been shown to be excessively expressed in the kidney, as well as cartilage and synovium in osteoarthritic patients [54]. Another factor implicated in chondrogenesis is *TRPS1*, another SNP reported in this study, which is a gene responsible for signalling of pathways involved in differentiation and proliferation, and the ureteric bud branching demonstrated in early renal organogenesis [55]. Although there is no obvious association between this factor and UI or OAB, soft tissue plays an important role in pelvic support, therefore, this is a conceivable mechanism through which urinary tract anatomical deterioration can generate LUTS.

Nevertheless, variants of β3 adrenoreceptor genes were not associated with OAB vs controls in the study included in this review; instead, these were correlated with PVR. This contrasts with a recent meta‐analysis of four studies which demonstrated moderate susceptibility to OAB conferred by a Trp64 Arg polymorphism of *ADRB3* [56]. The pathophysiology of detrusor activity is partially mediated by β3 adrenoreceptors through its role in parasympathetic control via catecholamines [57]; therefore, aberrances in gene function can lead to impairment of bladder relaxation mechanisms.

Whilst risk of bias assessments showed moderate bias stratification for several of the GWAS, as well as some unclear reporting environmental effect modifications, this review has described potential targets for personalised and stratified treatments of LUTS. The intricacies of the pathophysiology underpinning LUTS offer an encouraging avenue for future pharmacological management. *PGR*, progesterone receptor gene, yielded a 1.36 OR for BPH risk; indeed, it is a pharmacological target in BPH therapeutics [58]. This is also a factor often seen in prostate cancer patients, which was a significant confounding variable in our investigation, perhaps because they share similar pathogenetic risk factors. Based on considerable genome‐wide analysis, a multitude of potential biomarkers can nevertheless be put forward, in the context of genetic counselling, to estimate parameters such as probability of symptom progression.

Numerous study designs were included for analysis in this comprehensive investigation. Standardised piloted data extraction forms, and the SWiM procedure, were utilised with a prespecified quality assessment protocol. Furthermore, a wide variety of ages was included for review. A persistent misconception regarding LUTS is that it is a disease of the elderly (especially in reference to prostatism in elderly men); our review has demonstrated that phenotypic manifestations can occur as early as the third decade.

Limitations of this review include the relatively low numbers of included studies, reflective of the available evidence in this field. The studies are further limited by widespread inherent confounding. Prostate cancer was a main confounder in the identification phase of data synthesis, and a substantial proportion of genetic variability studies were excluded. Heterogeneity of LUTS definitions and phenotyping across included studies further complicated the comparisons between studies. To improve our understanding of genetic bearings on the pathophysiology of LUTS, further work is indicated to consolidate aspects of this investigation, such as lack of replication across studies. GWAS can be complemented by co‐twin control measures to better clarify if phenotypic association is mediated by genetics or environment [6], something that is perhaps overlooked in current LUTS studies.

In conclusion, twin studies, family studies, and GWAS have provided a multidimensional understanding of the causative factors underpinning LUTS. We have identified SNPs eliciting distinct LUTS phenotypes, and the level of inheritance in each of the LUTS categories. We have demonstrated familial clustering of specific LUTS phenotypes. Given the extensive genetic predisposition of LUTS that we have ascertained, additional synthesis and meta‐analysis of GWAS data are indicated, which will have implications for future clinical practice and decision‐making.

## Author Contributions

Lorcan Moore: Project development, literature search, data collection, data analysis, manuscript writing. Nicholas Raison: Project development, critically revising work, manuscript editing. Prokar Dasgupta: Critically revising work, manuscript editing. Arun Sahai: Critically revising work, manuscript editing. Sachin Malde: Critically revising work, manuscript editing, approval of final manuscript.

## Disclosure of Interests

None declared.

## Supporting information


**Table S1.** Data extraction table summarising studies assessing LUTS as a composite entity, with outcome measures and key findings from each report. Risk of Bias scores, calculated via Robins‐I assessment, are compiled in the final column.
**Table S2.** Data extraction table summarising studies assessing prostatic enlargement symptomatology, with key findings and risk of bias assessments.
**Table S3.** Data extraction table summarising studies assessing storage LUTS, OAB and SUI; with key findings and risk of bias assessments.
**Table S4.** ROBINS‐I assessment.
**Table S5.** JBI Checklist.
**Data S1.** Search terms.

## Appendix A

**Table A1:** List of SNPs and implicated genes.

SNP or loci	Associated gene	Normal function of gene	Associated with changes in LUTS prevalence compared to controls	LUTS subcategory	Study
‐34 T> C	*CYP17*	Adrenal and gonadal steroid biosynthesis	Yes	BPH	Abdullah et al. [12]
Trp64Arg Trp64Arg	*ADRB3* *ADRB3*	Implicated in detrusor relaxation pathophysiology	No Yes	UI (SUI, UUI, MUI) OAB	Aniulis et al. [13] Haga et al. [14]
T102C	*HTR2A*	Gene encoding serotonin 2A receptor: smooth muscle contraction	Yes	UI (SUI, UUI, MUI)	Aniulis et al. [13]
rs700518 rs10046	*CYP19A1*	Aromatase‐encoding gene implicated in testosterone metabolism	No	BPH	Berges et al. [15]
rs2740574	*CYP3A4*	Oxidation of testosterone	No	BPH	Berges et al. [15]
rs138724718	*MARCO*	Encodes scavenger receptors, implicated in immune system and inflammation	Yes	SUI	Cartwright et al. [16]
rs3998271	*EDN1*	Encodes potent vasoconstrictor	Yes	UUI	Cartwright et al. [16]
7p14.3	Novel miRNA Upstream Variant	Transcription factor binding site	Yes	UUI	Cartwright et al. [17]
7q34	*AGK*	Encodes mitochondrial membrane protein	Yes	UUI, SUI	Cartwright et al. [17]
14q22.3	*WDHD1*	Potentially involved in subcellular signal transduction and assembly	Yes	UUI	Cartwright et al. [17]
20q13.13	Transcription factor binding site		Yes	UUI	Cartwright et al. [17]
rs2736100 rs2736098	*TERT*	Maintenance of telomere ends and anti‐oncogenic properties	No No	BPH BPH	Cheng et al. [18]
rs12696304	*TERC*	Maintenance of telomere ends and chromosomal repair	No	BPH	Cheng et al. [18]
12q24.21: rs2555019 and rs8853	*TBX5, TBX3*	Associated with serum PSA levels and global embryonic development	Yes	BPH	Gudmundsson et al. [19]
13q14.3	*RNASEH2B*	Prevention of immune activation and nucleotide synthesis	Yes	BPH	Gudmundsson et al. [19]
20q13.33 rs17144046	*GATA5* *GATA3*	Encodes transcription factor; high expression in bladder and prostate tissues	Yes Yes	BPH BPH	Gudmundsson et al. [19] Na et al. [20]
rs10934853	*–*	Implicated in proliferation of bladder cells	Yes	LUTS composite	Helfand et al. [21]
rs445114	*–*	Implicated in proliferation of bladder cells	Yes	LUTS composite	Helfand et al. [21]
rs5945572	*–*	Implicated in proliferation of bladder cells	Yes	LUTS composite	Helfand et al. [21]
rs2710383	*SYN3*	Synapsin: implicated in release of neurotransmitters and synaptogenesis	Yes	BPH	Hellwege et al. [22]
rs534957	*GCLC*	Encodes rate‐limiting enzyme in glutathione synthesis	Yes	BPH	Hellwege et al. [22]
rs4239633	*UNC13A*	Expresion of variants associated with amyotrophic lateral sclerosis	Yes	BPH	Hellwege et al. [22]
rs36067435	*ELOVL*	Elongation of fatty acids	Yes	BPH	Hellwege et al. [22]
rs10786938	*SORCS1*	High expression within central nervous system, involved in neuropeptide receptor pathways	Yes	BPH	Hellwege et al. [22]
P346P	*MSR1*	Macrophase scavenger receptor: implicated in immune system and atherosclerosis	No	BPH	Hsing et al. [23]
rs103294	*LILRA3*	Innate immune system functioning and Class I MHC antigen binding	Yes	BPH	Jiao et al. [24]
rs8027714	*NPAP1*	Implicated in Prader‐Wili Syndrome	Yes	BPH	Li et al. [25]
rs8136152	*MPPED1*	Potentially modulates hydrolase activity	Yes	BPH	Li et al. [25]
rs1019233	*RBMS1*	Binding of ssDNA and ssRNA in gene transcription and apoptotic pathways	Yes	BPH	Li et al. [25]
rs1237696	*PGR*	Mediation of progesterone's physiological effects	Yes	BPH	Li et al. [25]
rs758937019‐C, rs758937019‐T	*TREK‐1*	Encodes pore domain potassium channels, implicated in bladder neck obstruction	Yes	LUTS composite	Nedumaran et al. [26]
8q23.3	*TRPS1*	Encoded protein suppresses expression of GATA‐related genes	No	UI	Penney et al. [27]
1p32.3	*DAB1*	Involved in neuronal migration during embryonic brain development	No	UI	Penney et al. [27]
rs7607995	*WDR54*	Regulation of cell differention via epidermal growth factor signalling pathway	Yes	SUI	Penney et al. [27]
rs4986791 rs115336889	*TLR4*	Implicated in innate immune system activation	Yes	BPH	Qiu et al. [28]
rs885786 rs6113	*SERPINA5*	Encoded protein inhibits proteins of haemostatic, thrombotic and inflammatory pathways	Yes*	SUI	Reischer et al. [29]
rs4293393 rs13333226 rs1333518	*UMOD*	Associated with protective mechanisms against urinary tract infections	No	SUI	Reischer et al. [29]
rs1800012	*COL1A1*	Encodes alpha chains of collagen for collagen synthesis	No	SUI	Reischer et al. [29]
rs1799750	*MMP1*	Involved in various tissue processes including remodelling, extracellular matrix breakdown and embryonic development	Yes*	SUI	Reischer et al. [29]
18q11	*ZFP521*	Potential upstream protein of neuronal fate determination	Yes	UUI	Richter et al. [30]
5p15	*ADAMTS16*	Modulates peptidase and metallopeptidase activity in cellular proliferation	Yes	UI	Richter et al. [30]
12q24	*CIT*	Cytokinesis and central nervous system development	Yes	UUI	Richter et al. [30]
Codon 219 Codon 384	*MLH1*	Tumour supressor gene: regulation of DNA damage surveillance	No No	BPH	Tanaka et al. [31]
rs10903035 rs11249006 rs12979860 rs8099917	*IL28Rα* >*IL28β*	Immune mediation and anti‐hyperplasia properties	Yes No	LUTS composite	Xiao et al. [32]

Descriptions of gene functions provided by individual studies and courtesy of *The GeneCards Suite* [33]. MUI, mixed urinary incontinence; OAB, overactive bladder; SNP, single‐nucleotide polymorphism; SUI, stress urinary incontinence; UI, urinary incontinence; UUI, urge urinary incontinence.

* Combination of *SERPINA5* and *MMP1* conferred statistically significant increased SUI risk, but significance was not achieved within distinct polymorphisms alone.
