# Supplementary material for: Inheritance patterns of lower urinary tract symptoms in adults: a systematic review
Source: BJU Int. 2024 Aug 26;135(2):192–203. doi: 10.1111/bju.16517 (PMC11745988; doi:10.1111/bju.16517)
Supplement: Supplementary file 1 — Table S1. Data extraction table summarising studies assessing LUTS as a composite entity, with outcome measures and key findings from each report. Risk of Bias scores, calculated via Robins‐I assessment, are compiled in the final column. Table S2. Data extraction table summarising studies assessing prostatic enlargement symptomatology, with key findings and risk of bias assessments. Table S3. Data extraction table summarising studies assessing storage LUTS, OAB and SUI; with key findings and risk of bias assessments. Table S4. ROBINS‐I assessment. Table S5. JBI Checklist. Data S1. Search terms. [file BJU-135-192-s001.docx]

**Supplementary Materials**

**Supplementary A: Search terms**

1. lower urinary tract symptoms.mp
2. prostatic hyperplasia.mp
3. urinary tract symptoms.mp
4. LUTS.mp
5. prostatism.mp
6. nocturia.mp
7. nycturia.mp
8. prostatic hypertrophy.mp
9. prostatic adenoma*.mp
10. urge incontinence.mp
11. urinary incontinence.mp
12. urinary stress.mp
13. urinary urge.mp
14. storage symptoms.mp
15. voiding symptoms.mp
16. urinary hesitancy.mp
17. urinary obstruction.mp
18. OAB.mp
19. overactive bladder.mp
20. stress incontinence.mp
21. 1 or 2 or 3 or 4 or 5 or 6 or 7 or 7 or 8 or 8 or 9 or 10 or 11 or 12 or 13 or 14 or 15 or 16 or 17 or 18 or 19 or 20
22. heritab*.mp
23. twin.mp
24. genomic.mp
25. genome-wide association stud*.mp
26. GWAS.mp
27. 22 or 23 or 24 or 25 or 26
28. 21 and 27
29. Remove duplicates from 28

***Supplementary table 1:*** Data extraction table summarising studies assessing LUTS as a composite entity, with outcome measures and key findings from each report. Risk of Bias scores, calculated via Robins-I assessment, are compiled in the final column.

| **Study information** | | | **Population** | | | | | **Results** | |
| --- | --- | --- | --- | --- | --- | --- | --- | --- | --- |
| Reference/ Country | | Gene symbol/ polymorphism (if applicable) of interest | Specific LUTS diagnosis | LUTS diagnostic tool(s) | Number, other demographic information | Male % | Mean age/ years | Key study findings, OR | ROB-  INS-I* |
| **GWAS** | | | | | | | | | |
|  | Haga et al., 2021/ Japan[12] | Variants of β_3_ adreno-receptor gene:  *TRP64ARG*  *ARG64ARG* | BPH  OAB | Ultrasound  PVR  Uroflowmetry  ICSS | Cases: 129  Controls: 247 | Cases: 100  Controls: 100 | Wild type: 67.00 ± 5.30  Cases: 67.00 ± 4.90 | PVR increased in variant type, and voiding time.  No association in β_3_ adrenoreceptor gene SNPs and LUTS.  OR: 0.870 |  |
|  | Helfand et al., 2013/ USA[20] | rs1571801  rs5945572  rs445114 | BPH  LUTS unspecified | AUA-SI Score  DRE | African-American men  1168 | 100 | 59 | Significance of SNPs and LUTS scores achieved at 3 loci.  SNPs  rs1571801: 1.29 OR  rs5945572: 1.23 OR  rs445114: 0.82 OR  (protective) |  |
|  | Na et al., 2017/ USA, China, Finland[21] | *GATA3*  rs17144046  rs943587 | BPH  Frequency  Urgency  Intermittency  Straining  Weak stream | IPSS  Prostate volumes  AUA symptom index  Uroflowmetry | REDUCE study: 1419 cases, 1684 controls/ 100  CLUE II: 568 cases, 568 controls/ 100  Finnish confirmation study: 485 cases, 475 controls/ 100 | REDUCE study: 100  CLUE II: 100  Finnish: 100 | REDUCE: cases 63.20 ± 5.80, controls 62.30 ± 6.10  CLUE II: cases 55.60 ± 10.60, controls 55.60 ± 10.50  Finnish: cases 66.00 ± 7.50, controls 65.60 ± 3.10 | 1 of 14 assessed loci reached genome-wide significance, rs17144046, in 3 independent populations. Conferred increased LUTS and BPH risk.  rs17144046  OR in confirmation study and meta-analysis: 1.41 (1.55 in confirmation study).  rs943587: OR 1.61 in Finnish confirmation study, but 1.11 with meta-analysis due to different OR directions in REDUCE and CLUE studies. (OR 0.88 and 0.75 respectively). |  |
|  | Xiao et al., 2015/ China[18] | *IL28-B/* rs12979860  rs8099917  *IL28-Rα/* rs10903035  rs11249006 | Frequency  Urgency  Intermittency  Straining  Weak stream | Medical history  Physical examination  Ultrasound  IPSS  Control exclusions: acute or chronic urinary retention, repeated haematuria | Cases: 233  Controls: 320 | Cases: 100  Controls: 100 | Cases: 70.59 ± 6.77  Controls: 41.96 ± 12.74 | Polymorphism in *IL28-Rα* gene associated with reduced LUTS severities; *IL-28B* polymorphism no effect.  Combined effects of all SNPs of *IL28-Rα/* had OR 0.429-0.553 = protective. |  |
| **Twin studies** | | | | | | | | | |
|  | Afari et al., 2016/ USA[15] |  | Frequency  Urgency  Intermittency  Straining  Weak stream  Nocturia | IPSS | 1002 MZ pairs  580 DZ pairs | MZ: 0  DZ: 0 |  | Genetic contributions to LUTS was 20-40%. IPSS >8 heritability was 37%.  Nocturia heritability 21%, straining 40%, UUI and frequency 34-36%. |  |
|  | Meikle et al., 1999/ USA[13] |  | Frequency  Urgency  Incomplete emptying  Straining  Weak stream | AUA Score  TRUS  DRE | MZ: 83 pairs  DZ: 83 pairs | MZ: 100  DZ: 100 | MZ: 55.00 ± 12.50  DZ: 54.30 ± 11.90 | Heritability accounted for 82.6% of symptom score variability in men over 50 years.  No evidence of heritability for weak stream and intermittency.  ***Heritability values:***  Frequency: 62%  Urgency: 43%  Straining: 75%  Incomplete emptying: 28%  Nocturia: 22% |  |
|  | Rohrmann et al., 2006/ USA[14] |  | Frequency  Urgency  Intermittency  Straining  Weak stream  Nocturia | IPSS | 3466 | 100 | 74.15 | Covariate analysis showed genetic factors accounted for 72% LUTS variability in moderate – severe cases.  OR 1.80 |  |
|  | Wennberg et al., 2011/ Sweden[17] |  | Frequency  Urgency  Nocturia  Incontinence  OAB | ICS definitions  UI criteria | 2275  MZ: 1392  DZ: 883 | 0*  Study included men and women; response rate too low for heritability analysis in male cohort | Range 20-46 | Higher genetic effects in symptoms of nocturia, incontinence, frequency, when adjusting for environmental (shared and individual factors) and incontinence due to pelvic floor weakness.  Nocturia, incontinence and frequency had genetic effects of 0.48, 0.51 and 0.40 respectively.  OAB (without concurrent UI, = OAB dry) had strongest environmental influences; genetic component was 0.04. All OAB categories combined were 0.10.  Secondary outcome: LUTS was much less prevalent in men. |  |
| **Case control, candidate gene association** | | | | | | | | | |
|  | Nedumaran et al., 2018/ USA[19] | 6 SNPs of *TREK-1:* specifically  rs758937019 | Incomplete emptying  Frequency  Urgency  Intermittency  Straining  Weak stream  Nocturia | AUA scores  Biopsy | Cases: 28  Controls: 30 | Cases: 100  Controls: 100 | N/A | CT and T polymorphism of rs758937019 genotype produced observable LUTS phenotype.  “Infinite” odds ratio for T and CT polymorphism.  Lower limit of 95% confidence intervals: 1.99 and 2.21 OR respectively. |  |
|  | Yue et al., 2019/ China[16] |  | BPH  Incomplete emptying  Frequency  Urgency  Intermittency  Straining  Weak stream  Nocturia | DRE  Ultrasound | Cases: 94  Controls: 106 | Cases: 100  Controls: 100 | Cases: 64.75 ± 11.75  Controls: 62.24 ± 12.36 | Heritability (*H_b_^2^)*, using Falconer method, was 40.48% for BPH.  Frequency, straining, incomplete emptying had strong heritability (*H*^2^ 71.37, 53.36, 43.28% respectively).  Nocturia, intermittent voiding, urgency, and weak stream were less heritable (*H*^2^ 19.12, 9.67, 5.67, 2.70% respectively) |  |
| AUA = American Urological Association, BFLUTS = Bristol female lower urinary tract symptoms questionnaire, BND = bladder neck descent, BPH = benign prostatic hyperplasia, DAN-PSS = Danish Prostatic Symptom Score, DRE = digital rectal examination, DZ = dizygotic, FLUTS = female lower urinary tract symptoms, GWAS = Genome-wide association study, ICD = International Classification of Diseases, ICIQ = International Consultation on Incontinence Questionnaire, IPSS = International Prostate Symptom Score, LUTS = lower urinary tract symptoms, MUI = mixed urinary incontinence, MZ = monozygotic, OAB = overactive bladder, OABSS = overactive bladder symptom score, OBND = oblique bladder neck descent, OR = odds ratio, PRS = polygenic risk score, PSA = prostate specific antigen, PVR = post-void residual, RR = relative risk, SNP = single nucleotide polymorphism, SUI = stress urinary incontinence, TRUS = transurethral ultrasound of prostate, UI = urinary incontinence, UUI = urge urinary incontinence, WHI = Women’s Health Initiative.  *Risk of bias assessment via ROBINS-I: **low, moderate, serious, critical.** | | | | | | | | | |

***Supplementary table 2:*** Data extraction table summarising studies assessing prostatic enlargement symptomatology, with key findings and risk of bias assessments.

| **Study information** | | | **Population** | | | **Results** | |
| --- | --- | --- | --- | --- | --- | --- | --- |
| Reference/ Country | | Gene symbol/ polymorphism (if applicable) of interest | LUTS diagnostic tool(s) | Number | Mean age/ years | Key study findings | ROB-  INS-I* |
| **GWAS** | | | | | | | |
|  | Abdullah et al., 2018/ Iraq[31] | *CYP17*  34 T > C | PSA levels  Symptom questionnaire  Biopsy | Cases: 50  Controls: 50 | No mean provided.  Cases: 45-80  Controls: 45-60 | T alleles more likely to confer BPH symptoms than those with C alleles, with pattern of codominance most significant. |  |
|  | Cheng et al., 2020/ China[33] | *TERT*  *TERC* | Ultrasound | Cases: 490  Controls: 322 | Cases: 51.4 ± 8.2  Controls: 52.3 ± 8.0 | BPE patients exhibited shorter LTL than controls.  Polymorphisms at *TERT* and *TERC* loci (telomerase holoenzyme genes) were not associated with BPH.  **LTL**: Cases  0.899 ± 0.322  Controls: 1.231 ± 0.532 |  |
|  | Gudmundsson et al., 2018/ Iceland, UK[29] | *15 loci* | Men undergoing BPH treatment with G04C group drugs | Iceland: 9443 cases  104,000 controls  UK: 11,178 cases    176.541 controls | 71 | 15 variants were associated with PSA levels in BPH patients.  8.6% increase in PSA for every standard deviation increase in PRS, in BPH, with joint-conditioning of PCa. Without conditioning: 12.9% increase. |  |
|  | Hellwege et al., 2019/ USA[25] | *SYN3*  *GCLC*  *UNC13A*  *ELOVL*  *SORCS1* | BPH | Cases: 2656  Controls: 7763 | Cases: 68.88  Controls: 61.45 | Demonstrated heritability of BPH, with BPH-susceptible loci on chromosomes 5 and 6 particularly.  H^2^ values: 0.65 (cohort 1)  *SYN3:* 0.63  *GCLC:* 1.26  *UNC13A:* 1.25  *ELOVL:* 0.83  *SORCS1:* 1.23 (whites only) |  |
|  | Li et al., 2021/ UK[24] | rs8027714 (*NPAP1)*  rs8136152  (MPPED*1)*  rs1019233  (*RBMS1*)  rs1237696  (*PGR*) | BPH | Cases: 1942  Controls: 4730 | UK Biobank: age between 40-69 | With validation cohort, 4 SNPs achieved genome-wide significance, showing association with risk of BPH. Two SNPs conferred reduced risk, two conferred increased risk.  OR:  rs8027714: 0.66  rs8136152: 0.72  rs1019233: 1.29  rs1237696: 1.36 |  |
|  | Na et al., 2017/ USA, China, Finland[21] | *GATA3*  rs17144046  rs943587 | IPSS  Prostate volumes  AUA symptom index  Uroflowmetry | REDUCE study: 1419 cases, 1684 controls/  CLUE II: 568 cases, 568 controls  Finnish: 485 cases, 475 controls | REDUCE: cases 63.20 ± 5.80, controls 62.30 ± 6.10  CLUE II: cases 55.60 ± 10.60, controls 55.60 ± 10.50  Finnish: cases 66.00 ± 7.50, controls 65.60 ± 3.10 | Only 1 of 14 assessed loci reached genome-wide significance, rs17144046, in 3 independent populations. Conferred increased LUTS and BPH risk.  rs943587 did not reach genome-wide significance after meta-analysis.  rs17144046  OR in confirmation study and meta-analysis: 1.41 (1.55 in confirmation study).  rs943587: 1.61 in confirmation study, but 1.11 with meta-analysis. |  |
|  | Qiu et al., 2019/ China[23] | *TLR4* | IPSS  Maximum flow rate  Prostate volumes  PSA | 501 BPH  964 controls | Cases: 71.43 ± 5.31  Controls: 70.65 ± 6.43 | SNPs within *TLR4* were useful in predicting both prognosis and risk of BPH.  Specifically, SNPs in rs4986791 and rs115336889 conferred increased risk of BPH.  OR:  rs4986791: 2.96  rs115336889: 2.50 |  |
| **Twin studies** | | | | | | | |
|  | Gasperi et al., 2019/ USA[32] |  | BPH | 6824 individuals  Enlarged prostate 18.8% total. | 61.10  Range 53-73 years | Enlarged prostate/ BPH had heritability of 20% using best-fitting reduced common pathway model. |  |
|  | Partin et al., 1994[34] |  | Questionnaire | 256 twin pairs | 64 ± 3 | Proband wise concordance rates were 25.7% and 8.5% for MZ and DZ twins respectively.  Using ACE model, genetic factors accounted for 49% of variance in responsibility of benign prostatic disease.  RR: 3.3 |  |
| **Case control, candidate gene association** | | | | | | | |
|  | Berges et al., 2009/ Germany[28] | *CYP19A1*  *CYP3A4* | BPH  IPSS  TRUS  USS  PSA levels | 392 | 65.40 ± 7.00 | No significant different in IPSS and Q_max_ across homozygous and heterozygous variants of *CYP19A1.* Men carrying rs10046 heterozygous genotype had higher PSA levels (2.00 ± 0.10 ng/ml vs wildtype 1.70 ± 0.20 ng/ml, P = 0.012).  No association between *CYP3A4* polymorphism and IPSS (non-significant p-value) or Q_max_. |  |
|  | Hsing et al., 2007/ China[27] | *MSR1* | ICD-9  Blood sample | Cases: 130  Controls: 155 | Cases: 68.60 ± 6.00  Controls: 70.80 ± 8.10 | No evidence of clear link between BPH risk and *MSR1* variants.  OR 1.9 for haplotype (C – (–) – A – A); p value 0.26 so not statistically significant. |  |
|  | Jiao et al., 2013/ China[22] | *LILRA3* | IPSS  Ultrasound (PVR)  DRE  PSA level | Cases: 426  Controls: 1028 | Cases: 71.90 ± 7.90  Controls: 61.20 ± 9.00 | *LILRA3*, specifically rs103294 SNP, associated with increased BPH risk.  OR 1.34 for cases with C risk allele; 1.51 for cases <72 years old. |  |
|  | Pearson et al., 2003/ USA[30] |  | Physical examination  Questionnaire | Father of proband:  Cases: 132  Controls: 141  Brother of proband:  Cases: 198  Controls: 207 | Father:  Case: 63.90 ± 9.10  Control: 68.90 ± 12.10  Brother:  Case: 62.40 ± 7.70  Control: 57.60 ± 13.00 | Demonstrated importance of family history in incidence of BPH.  Mode of inheritance most likely is autosomal dominant or codominant.  OR 2.11 for fathers of those with BPH.  OR 3.52 for brothers of those with BPH.  OR for BPH:  1 1^st^ degree relative affected = 1.72  2 1^st^ degree relatives affected = 4.74. |  |
|  | Tanaka et al., 2010/ Japan[26] | *MLH1*  Codons 219 and 384 | PSA levels | Cases: 134  Controls: 131 | Cases: 73.20 ± 0.70  Controls: 67.30 ± 1.00 | No differences between controls and cases in codons 219 or 384.  Concluded that these codons are not implicated in BPH development.  Codon 219: 0.88  Codon 384: 0.81 |  |
| AUA = American Urological Association, BND = bladder neck descent, BPH = benign prostatic hyperplasia, DAN-PSS = Danish Prostatic Symptom Score, DRE = digital rectal examination, DZ = dizygotic, GWAS = Genome-wide association study, ICD = International Classification of Diseases, IPSS = International Prostate Symptom Score, LUTS = lower urinary tract symptoms, MZ = monozygotic, OR = odds ratio, PRS = polygenic risk score, PSA = prostate specific antigen, Q_max_ = maximum uroflow (mL/s), RR = relative risk, SNP = single nucleotide polymorphism, SUI = stress urinary incontinence, TRUS = transurethral ultrasound of prostate.  *Risk of bias assessment via ROBINS-I: **low, moderate, serious, critical.** | | | | | | | |

***Supplementary table 3:*** Data extraction table summarising studies assessing storage LUTS, OAB and SUI; with key findings and risk of bias assessments.

| **Study information** | | | | **Population** | | | | | **Results** | |
| --- | --- | --- | --- | --- | --- | --- | --- | --- | --- | --- |
| Reference/ Country | | Gene symbol/ polymorphism (if applicable) of interest | | Specific LUTS diagnosis | LUTS diagnostic tool(s) | Number | Male % | Mean age/ years | Key study findings | RO-BIN-  S-I* |
| **OAB** | | | | | | | | | | |
| **GWAS** | | | | | | | | | | |
|  | Funada et al., 2017/ Japan[35] | *ADAMTS16*  *SLC18A1*  *MATN1*  *IRS1*  *BBOX1*  *SOX5* | | OAB | OABSS | 4645 controls  549 OAB cases | 32.70 | Cases: 63 median  Controls: 54 median | OAB wet associated with rs4467538, but not in replication study.  No association of *ADAMTS16* to any type of OAB.  OAB heritability 0.027 |  |
| **Twin studies** | | | | | | | | | | |
|  | Wennberg et al., 2011/ Sweden[17] |  | | OAB | ICS definitions  UI criteria | 2275  MZ: 1392  DZ: 883 | 0 | N/A  Range 20-46 | OAB (without concurrent UI, = OAB dry) had strongest environmental influences; genetic component was 0.04. All OAB categories combined were 0.10. |  |
| **Nocturia, frequency** | | | | | | | | | | |
| **Twin studies** | | | | | | | | | | |
|  | Afari et al., 2016/ USA[15] |  | | Nocturia | IPSS | 1002 MZ pairs  580 DZ pairs | MZ 0  DZ 0 | 50.20 | Nocturia heritability 21%, straining 40%, UUI and frequency 34-36%. |  |
|  | Meikle et al., 1999/ USA[13] |  | | Nocturia | AUA Score  TRUS  DRE | MZ: 83 pairs  DZ: 83 pairs | MZ 100  DZ 100 | MZ: 55.00 ± 12.50  DZ: 54.30 ± 11.90 | No evidence of any heritability for weak stream and intermittency. |  |
|  | Wennberg et al., 2011/ Sweden[17] |  | | Nocturia  Frequency | ICS definitions  UI criteria | 2275  MZ: 1392  DZ: 883 | 0 | N/A  Range 20-46 | Higher genetic effects in symptoms of nocturia, incontinence, frequency, when adjusting for environmental (shared and individual factors) and incontinence due to pelvic floor weakness.  Nocturia, incontinence and frequency had genetic effects of 0.48, 0.51 and 0.40 respectively. |  |
| **Case control, candidate gene association** | | | | | | | | | | |
|  | Yue et al., 2019/ China[16] |  | | Nocturia  Frequency | DRE  Ultrasound | Cases: 94  Controls: 106 | Cases: 100  Controls: 100 | Cases: 64.75 ± 11.75  Controls: 62.24 ± 12.36 | Frequency had strong heritability (*H*^2^ 71.37).  Nocturia was less heritable (*H*^2^ 19.12). |  |
| **UI** | | | | | | | | | | |
| **GWAS** | | | | | | | | | | |
|  | Cartwright et al. 2021/ UK, Finland[38] | | *MARCO*  rs138724718  *EDN1*  rs3998271 | SUI  UUI | BFLUTS  Questionnaire  DAN-PSS questionnaire  Bladder biopsy | 8798  Replication cohort: 4069 | 0  Replication cohort: 0 | 50.30 | *MARCO* associated with SUI, *EDN1* associated with UUI.  rs3998271: discovery cohort 1.85, replication 1.73  rs3998271: discovery cohort 1.70. replication 1.55 |  |
|  | Cartwright et al., 2014/ UK[39] | | Locus 7p14.3  Locus 7q34 *(AGK)* Locus 14q22.3 *(WDHD1)*  Locus 20q13.13 | SUI  UUI | BFLUTS questionnaire | 8997 | 0 | 45 | Common genetic predisposition and susceptibility to SUI and UUI.  Intron variant of *AGK* associated with both SUI and UUI. Other SNPs showed genome wide significance for SUI only.  OR:  7p: 4.27  7q: 3.21  14q: 0.45  20q: 8.48 |  |
|  | Penney et al., 2020/ USA[36] | | *TRPS1*  *DAB1*  *WDR54* | SUI  UUI  MUI | Questionnaire | 6120 cases  4811 controls | Cases: 0  Controls: 0 | Cases: median 61  Controls: median 54 | UI associated with polymorphisms near *TRPS1* and *DAB1* loci.  No genome-wide associations with regards to UI subtypes: UUI, SUI, MUI.  *WDR54* genome wide significant for SUI risk adjusting for known risk factors.  8 SNPs associated with UI located in  *TRPS1* and *DAB1* loci (p <5 x 10^-8^). |  |
|  | Richter et al., 2015/ USA[40] | | *ZFP521*  *ADAMTS16*  *CIT* | UUI | WHI questionnaire | 4894  2241 UUI | 0 | No mean | Polymorphisms in *ZFP521*  and *CIT* were associated with UUI phenotype.  *ADAMTS16*  associated with slight increase in UI phenotype risk. |  |
| **Twin studies** | | | | | | | | | | |
|  | Afari et al., 2016/ USA[15] | |  | UUI | IPSS | 1002 MZ pairs  580 DZ pairs | MZ 0  DZ 0 | 50.20 | Genetic contributions to LUTS was 20-40%. IPSS >8 heritability was 37%.  Nocturia heritability 21%, straining 40%, UUI and frequency 34-36%. |  |
|  | Altman et al., 2008/ Sweden[41] | |  | SUI | Swedish Inpatient Registry | 6752 MZ  10134 DZ | MZ 0  DZ 0 | N/A  48-81 age range | Genetic effects accounted for 41% of variations in liability in SUI cases.  *Tetrachoric correlations:*  0.61 in MZ  0.43 in DZ |  |
|  | Dietz et al., 2005/ Australia[45] | |  | BND  OBND | Translabial ultrasound | 46 MZ pairs  24 DZ pairs  38 non-twins | MZ 0  DZ 0  Non-twins 0 | N/A  18-24 age range | OBND more heritable influenced than BND: but large confidence intervals so could not rule out influences of the environment.  ACE modelling: 59% variance in OBND was due to additive genes, 51% in BND. |  |
|  | Nguyen et al., 2008/ USA[43] | |  | SUI | Questionnaire | MZ: 765 pairs  DZ: 117 pairs | MZ 0  DZ 0 | No mean | Environmental factors contributed 77.6% of variance in SUI; genetics only accounted for 1.49% when using ACE model. |  |
|  | Rohr et al., 2004/ Denmark[44] | |  | SUI  MUI  Composite UI | Questionnaire | 2336  MZ: 1096  DZ: 1240 | 0 | No mean | UI and mixed incontinence higher in MZ for both 46-68 and 70-94 age groups.  Stress incontinence not statistically significant between MZ and DZ.  Heritability for UI 42% and 49% in respective age group; mixed incontinence 27% and 55%. Stress incontinence best explained by familial and individual environment models.  OR:  46-68: MZ 0.51 UI, DZ -0.22 UI.  70-94: MZ 0.50 UI, DZ 0.28 UI. |  |
| **Case control, candidate gene association** | | | | | | | | | | |
|  | Aniulis et al., 2021/ Lithuania[37] | | *HTR2A*  *ADRB3* | UUI | ICIQ-FLUTS questionnaire | Cases: 110  Controls: 105 | Cases: 0  Controls: 0 | Cases:  UUI: 61.80  ± 7.30  Controls: 52.40 ± 8.20 | No difference observed in cases and controls in *ADRB3*.  *HTR2A* CC homozygosity associated with UUI.  3.06 OR in CC genotype of *HTR2A* in UUI. |  |
|  | Reischer et al., 2020/ Austria[42] | | *SERPINA5*  *UMOD*  *COL1A1*  *MMP1* | SUI | Questionnaire  Positive provocation test | 38  19 cases, 19 age-matched controls | 0 | Cases: 49.20 ± 9.60  Controls: 49.60 ± 9.70 | Combined presence of SNPs in *SERPINA5*, *MMP1* were associated with SUI.  Other genes reported no significant differences in cases and controls. |  |
| AUA = American Urological Association, BFLUTS = Bristol female lower urinary tract symptoms questionnaire, BND = bladder neck descent, BPH = benign prostatic hyperplasia, DAN-PSS = Danish Prostatic Symptom Score, DRE = digital rectal examination, DZ = dizygotic, FLUTS = female lower urinary tract symptoms, GWAS = Genome-wide association study, ICD = International Classification of Diseases, ICIQ = International Consultation on Incontinence Questionnaire, IPSS = International Prostate Symptom Score, LUTS = lower urinary tract symptoms, MUI = mixed urinary incontinence, MZ = monozygotic, OAB = overactive bladder, OABSS = overactive bladder symptom score, OBND = oblique bladder neck descent, OR = odds ratio, PRS = polygenic risk score, PSA = prostate specific antigen, PVR = post-void residual, RR = relative risk, SNP = single nucleotide polymorphism, SUI = stress urinary incontinence, TRUS = transurethral ultrasound of prostate, UI = urinary incontinence, UUI = urge urinary incontinence, WHI = Women’s Health Initiative.  *Risk of bias assessment via ROBINS-I: **low, moderate, serious, critical.** | | | | | | | | | | |

***Supplementary Table 4:*** ROBINS-I assessment

| **Study** | **Pre-intervention domains** | | **At-intervention domain** | **Post-intervention domains** | | | | **Overall risk of bias** |
| --- | --- | --- | --- | --- | --- | --- | --- | --- |
| Study, year/ country | Bias due to baseline confounding | Bias due to selection of participants | Bias due to classification of interventions | Bias due to deviation from intended interventions | Bias to due missing data | Bias in measurement of outcomes | Bias in selection of the reported result | Low/ moderate/ serious/ critical |
| Abdullah et al., 2018/ Iraq[46] | **moderate** | **serious** | **low** | **low** | **moderate** | **low** | **low** | **serious** |
| Afari et al., 2016/ USA[15] | **moderate** | **low** | **low** | **low** | **low** | **low** | **low** | **moderate** |
| Altman et al., 2008/ Sweden[41] | **moderate** | **low** | **low** | **low** | **low** | **low** | **low** | **moderate** |
| Aniulis et al., 2021/ Lithuania[47] | **low** | **low** | **low** | **low** | **low** | **low** | **low** | **low** |
| Berges et al., 2009/ Germany[28] | **moderate** | **low** | **low** | **low** | **low** | **low** | **moderate** | **moderate** |
| Cartwright et al. 2021/ UK, Finland[38] | **low** | **low** | **low** | **low** | **low** | **low** | **low** | **low** |
| Cartwright et al., 2014/ UK[48] | **moderate** | **moderate** | **low** | **low** | **serious** | **low** | **low** | **serious** |
| Cheng et al., 2020/ China[33] | **serious** | **low** | **low** | **low** | **low** | **low** | **low** | **serious** |
| Dietz et al., 2005/ Australia[45] | **moderate** | **low** | **low** | **low** | **low** | **low** | **low** | **moderate** |
| Funada et al., 2017/ Japan[35] | **low** | **low** | **low** | **low** | **moderate** | **low** | **moderate** | **moderate** |
| Gasperi et al., 2019/ USA[32] | **moderate** | **low** | **low** | **low** | **low** | **low** | **moderate** | **moderate** |
| Gudmundsson et al., 2018/ Iceland, UK[29] | **low** | **low** | **low** | **low** | **low** | **low** | **low** | **low** |
| Haga et al., 2021/ Japan[49] | **moderate** | **low** | **moderate** | **low** | **low** | **low** | **low** | **moderate** |
| Helfand et al., 2013/ USA[50] | **low** | **moderate** | **low** | **low** | **serious** | **low** | **moderate** | **serious** |
| Hellwege et al., 2019/ USA[25] | **moderate** | **low** | **low** | **low** | **low** | **low** | **low** | **moderate** |
| Hsing et al., 2007/ China[27] | **moderate** | **moderate** | **low** | **low** | **low** | **low** | **low** | **moderate** |
| Jiao et al., 2013/ China[22] | **low** | **low** | **low** | **low** | **low** | **low** | **moderate** | **moderate** |
| Li et al., 2021/ UK[24] | **moderate** | **low** | **low** | **low** | **low** | **moderate** | **low** | **moderate** |
| Meikle et al., 1999/ USA[13] | **moderate** | **low** | **moderate** | **low** | **moderate** | **low** | **low** | **moderate** |
| Na et al., 2017/ USA, China, Finland[21] | **moderate** | **low** | **low** | **low** | **moderate** | **low** | **low** | **moderate** |
| Nedumaran et al., 2018/ USA[51] | **serious** | **low** | **low** | **low** | **low** | **serious** | **low** | **serious** |
| Nguyen et al., 2008/ USA[43] | **moderate** | **low** | **moderate** | **low** | **moderate** | **moderate** | **low** | **moderate** |
| Partin et al., 1994[34]/ USA | **serious** | **moderate** | **moderate** | **low** | **low** | **low** | **low** | **serious** |
| Pearson et al., 2003/ USA[52] | **moderate** | **low** | **moderate** | **low** | **low** | **low** | **low** | **moderate** |
| Penney et al., 2020/ USA[36] | **low** | **moderate** | **low** | **low** | **moderate** | **low** | **low** | **moderate** |
| Qiu et al., 2019/ China[53] | **low** | **low** | **low** | **low** | **moderate** | **low** | **moderate** | **moderate** |
| Reischer et al., 2020/ Austria[42] | **low** | **low** | **low** | **low** | **low** | **low** | **low** | **low** |
| Richter et al., 2015/ USA[40] | **moderate** | **low** | **low** | **low** | **low** | **low** | **low** | **moderate** |
| Rohr et al., 2004/ Denmark[44] | **serious** | **low** | **low** | **low** | **moderate** | **low** | **low** | **serious** |
| Rohrmann et al., 2006/ USA[14] | **low** | **low** | **low** | **low** | **low** | **low** | **low** | **low** |
| Tanaka et al., 2010/ Japan[54] | **serious** | **low** | **low** | **low** | **moderate** | **low** | **low** | **serious** |
| Wennberg et al., 2011/ Sweden[17] | **low** | **moderate** | **low** | **low** | **low** | **low** | **low** | **moderate** |
| Xiao et al., 2015/ China[18] | **low** | **serious** | **moderate** | **low** | **low** | **low** | **low** | **serious** |
| Yue et al., 2019/ China[16] | **moderate** | **low** | **moderate** | **low** | **low** | **low** | **low** | **moderate** |

***Supplementary Table 5:*** JBI Checklist

| **Study** | **Q1** | **Q2** | **Q3** | **Q4** | **Q5** | **Q6** | **Q7** | **Q8** | **Q9** | **Q10** | **% Yes** | **Risk score** |
| --- | --- | --- | --- | --- | --- | --- | --- | --- | --- | --- | --- | --- |
| Abdullah et al., 2018/ Iraq[46] | 🗸 | 🗸 | ? | 🗸 | 🗴 | 🗸 | 🗴 | 🗸 | 🗸 | 🗸 | 70 |  |
| Afari et al., 2016/ USA[15] | 🗸 | 🗸 | 🗸 | 🗸 | 🗸 | 🗸 | 🗴 | 🗸 | 🗸 | 🗸 | 90 |  |
| Altman et al., 2008/ Sweden[41] | 🗸 | 🗸 | 🗸 | 🗸 | 🗸 | 🗸 | 🗴 | 🗸 | 🗸 | 🗸 | 90 |  |
| Aniulis et al., 2021/ Lithuania[47] | 🗸 | 🗸 | 🗸 | 🗸 | 🗸 | 🗸 | 🗸 | 🗸 | 🗸 | 🗸 | 100 |  |
| Berges et al., 2009/ Germany[28] | 🗸 | 🗸 | 🗸 | 🗸 | 🗸 | 🗸 | 🗴 | 🗸 | 🗸 | 🗸 | 90 |  |
| Cartwright et al. 2021/ UK, Finland[38] | 🗸 | 🗸 | 🗸 | 🗸 | 🗸 | 🗸 | 🗸 | 🗸 | 🗸 | 🗸 | 100 |  |
| Cartwright et al., 2014/ UK[48] | ? | ? | 🗸 | 🗸 | 🗸 | 🗴 | 🗴 | 🗸 | 🗸 | ? | 50 |  |
| Cheng et al., 2020/ China[33] | 🗸 | 🗸 | ? | 🗸 | 🗸 | 🗴 | 🗴 | 🗸 | 🗸 | 🗸 | 70 |  |
| Dietz et al., 2005/ Australia[45] | 🗸 | 🗸 | 🗸 | 🗸 | 🗸 | 🗴 | 🗴 | 🗸 | 🗸 | 🗸 | 80 |  |
| Funada et al., 2017/ Japan[35] | 🗸 | 🗸 | 🗸 | 🗸 | 🗸 | 🗸 | 🗴 | 🗸 | 🗸 | 🗸 | 90 |  |
| Gasperi et al., 2019/ USA[32] | 🗸 | 🗸 | 🗸 | 🗸 | 🗸 | 🗴 | 🗴 | 🗸 | 🗸 | 🗸 | 80 |  |
| Gudmundsson et al., 2018/ Iceland, UK[29] | 🗸 | 🗸 | 🗸 | 🗸 | 🗸 | 🗸 | 🗴 | 🗸 | 🗸 | 🗸 | 90 |  |
| Haga et al., 2021/ Japan[49] | 🗸 | 🗸 | 🗸 | 🗸 | 🗸 | 🗸 | 🗴 | 🗴 | 🗸 | 🗸 | 80 |  |
| Helfand et al., 2013/ USA[50] | ? | ? | 🗸 | 🗸 | 🗸 | 🗸 | 🗴 | ? | 🗸 | ? | 50 |  |
| Hellwege et al., 2019/ USA[25] | 🗸 | 🗸 | 🗸 | 🗸 | 🗸 | 🗸 | 🗴 | 🗸 | 🗸 | 🗸 | 90 |  |
| Hsing et al., 2007/ China[27] | 🗴 | 🗸 | 🗸 | 🗸 | 🗸 | 🗸 | 🗴 | 🗸 | 🗸 | 🗸 | 80 |  |
| Jiao et al., 2013/ China[22] | 🗸 | 🗸 | 🗸 | 🗸 | 🗸 | 🗸 | 🗸 | 🗸 | 🗸 | 🗸 | 100 |  |
| Li et al., 2021/ UK[24] | ? | ? | 🗸 | 🗸 | 🗸 | 🗸 | 🗴 | 🗸 | 🗸 | 🗸 | 70 |  |
| Meikle et al., 1999/ USA[13] | 🗴 | 🗴 | 🗸 | 🗸 | 🗸 | 🗸 | 🗴 | 🗸 | 🗸 | ? | 60 |  |
| Na et al., 2017/ USA, China, Finland[21] | 🗸 | 🗸 | 🗸 | 🗸 | 🗸 | 🗸 | 🗴 | 🗸 | 🗸 | 🗸 | 90 |  |
| Nedumaran et al., 2018/ USA[51] | ? | ? | 🗸 | 🗸 | 🗸 | 🗴 | 🗴 | 🗸 | 🗸 | 🗸 | 60 |  |
| Nguyen et al., 2008/ USA[43] | 🗸 | 🗸 | 🗸 | 🗴 | 🗸 | ? | 🗸 | 🗸 | 🗸 | 🗸 | 80 |  |
| Partin et al., 1994[34]/ USA | 🗸 | 🗸 | 🗸 | 🗸 | 🗴 | ? | 🗸 | 🗸 | 🗸 | ? | 70 |  |
| Pearson et al., 2003/ USA[52] | 🗸 | 🗸 | ? | 🗸 | 🗸 | ? | 🗴 | 🗸 | 🗸 | 🗸 | 70 |  |
| Penney et al., 2020/ USA[36] | 🗸 | 🗴 | 🗸 | 🗸 | 🗸 | 🗸 | 🗸 | 🗸 | 🗸 | 🗸 | 90 |  |
| Qiu et al., 2019/ China[53] | 🗸 | 🗸 | 🗴 | 🗸 | 🗸 | 🗸 | 🗸 | 🗸 | 🗸 | 🗸 | 90 |  |
| Reischer et al., 2020/ Austria[42] | 🗸 | 🗸 | 🗸 | 🗸 | 🗴 | 🗸 | 🗸 | 🗸 | 🗸 | 🗸 | 90 |  |
| Richter et al., 2015/ USA[40] | 🗸 | 🗸 | 🗸 | 🗸 | 🗸 | 🗸 | 🗴 | 🗸 | 🗸 | 🗸 | 90 |  |
| Rohr et al., 2004/ Denmark[44] | ? | ? | 🗸 | 🗸 | 🗸 | 🗴 | 🗴 | 🗸 | 🗸 | 🗸 | 60 |  |
| Rohrmann et al., 2006/ USA[14] | 🗸 | 🗸 | 🗸 | 🗸 | 🗸 | 🗸 | 🗸 | 🗸 | 🗸 | 🗸 | 100 |  |
| Tanaka et al., 2010/ Japan[54] | 🗸 | 🗸 | 🗸 | ? | ? | 🗴 | 🗴 | 🗸 | 🗸 | 🗸 | 60 |  |
| Wennberg et al., 2011/ Sweden[17] | 🗸 | 🗸 | 🗸 | 🗸 | 🗸 | ? | ? | 🗸 | 🗸 | 🗸 | 80 |  |
| Xiao et al., 2015/ China[18] | 🗴 | 🗴 | ? | 🗸 | 🗸 | 🗸 | ? | 🗸 | 🗸 | 🗸 | 60 |  |
| Yue et al., 2019/ China[16] | 🗸 | 🗸 | 🗸 | 🗸 | 🗸 | 🗸 | 🗴 | 🗴 | 🗸 | 🗸 | 80 |  |
| ***Q1***: Groups comparable other than presence of disease in cases or disease absence in controls? ***Q2:*** Cases and controls appropriately matched? ***Q3:*** Same criteria used to identify cases and controls? ***Q4:*** Standard, valid, and reliable way of measuring exposure? ***Q5:*** Exposure measured in same way for cases and controls? ***Q6:*** Identified confounding factors? ***Q7:*** Stated strategies to deal with confounding factors? ***Q8:*** Standard, valid, and reliable way of assessing control and cases outcomes? ***Q9:*** Exposure period of interest long enough to be meaningful? ***Q10:*** Appropriate statistical analysis method?  Thresholds: 80-100 = **low bias**, 60-70 = **moderate bias**, <60 = **serious bias**. | | | | | | | | | | | | |
